# Small Molecule Inhibitor of Formin Homology 2 Domains (SMIFH2) Reveals the Roles of the Formin Family of Proteins in Spindle Assembly and Asymmetric Division in Mouse Oocytes

**DOI:** 10.1371/journal.pone.0123438

**Published:** 2015-04-02

**Authors:** Hak-Cheol Kim, Yu-Jin Jo, Nam-Hyung Kim, Suk Namgoong

**Affiliations:** Department of Animal Sciences, Chungbuk National University, Cheong-Ju, ChungBuk, Republic of Korea; Institute of Zoology, Chinese Academy of Sciences, CHINA

## Abstract

Dynamic actin reorganization is the main driving force for spindle migration and asymmetric cell division in mammalian oocytes. It has been reported that various actin nucleators including Formin-2 are involved in the polarization of the spindle and in asymmetric cell division. In mammals, the formin family is comprised of 15 proteins. However, their individual roles in spindle migration and/or asymmetric division have not been elucidated yet. In this study, we employed a newly developed inhibitor for formin family proteins, small molecule inhibitor of formin homology 2 domains (SMIFH2), to assess the functions of the formin family in mouse oocyte maturation. Treatment with SMIFH2 during *in vitro* maturation of mouse oocytes inhibited maturation by decreasing cytoplasmic and cortical actin levels. In addition, treatment with SMIFH2, especially at higher concentrations (500 μM), impaired the proper formation of meiotic spindles, indicating that formins play a role in meiotic spindle formation. Knockdown of the mDia2 formins caused a similar decrease in oocyte maturation and abnormal spindle morphology, mimicking the phenotype of SMIFH2-treated cells. Collectively, these results suggested that besides Formin-2, the other proteins of the formin, including mDia family play a role in asymmetric division and meiotic spindle formation in mammalian oocytes.

## Introduction

During meiosis I, mammalian oocytes undergo transitions via various stages of cell cycle[1]. When immature oocytes start to mature by hormonal stimulation, germinal vesicle breakdown (GVBD) happen and meiotic spindle formation is completed at metaphase I (MI) stage. One of the hallmarks distinguishing meiosis I in oocytes from mitosis in somatic cells are asymmetric division[2]. Particularly the formation of the meiotic spindle and the migration of the spindle to near the cortex are crucial events for asymmetric division[3, 4] and it followed by anaphase I to telophase I transition and matured oocyte are arrested in metaphase II (MII) stage and progress of further cell cycle was arrested until fertilization by sperm.

Dynamic actin reorganization is a main driving force for intracellular movements of the meiotic spindle[5, 6]. Various actin nucleators, including Formin-2[7–9], Spire[10] and the Arp2/3 complex[11, 12], play essential roles in the asymmetric migration of the spindle by promoting the formation of new actin filaments. In addition to actin nucleators, nucleation promoting factors (NPF) such as N-WASP[13], WAVE2[14]^,^ [15], WASH[16] or JMY[17] are involved in the asymmetric division of oocytes by activating the Arp2/3 complex, thereby promoting actin polymerization. In addition, actin-binding proteins including tropomyosin[18] and actin capping protein[19] play important roles in oocyte maturation by regulating the stability and growth of the actin filaments. Because there are more than 100 different types of actin-binding proteins in mammals, most of them playing crucial roles in the formation and maintenance of the actin cytoskeleton[20, 21], many actin-binding proteins have been hypothesized to have an important function in oocyte maturation. However, the exact roles of many actin-binding proteins including actin nucleators in the asymmetric division of oocytes have not been elucidated till date.

Besides Formin-2, encoded by the gene *Fmn2* in humans and mice, mammals have 14 other formin family proteins[22] including the mDia (a mammalian homolog of *Drosophila* diaphanous) subfamily, which play various important roles in cytokinesis[23, 24], the formation of fillopodia[25], the maintenance of cortex integrity[26], and mitochondrial fission[27]. Previous studies on the function of formins in oocyte maturation have been focused on Formin-2. Mutations in the *Fmn2* gene cause infertility in mouse[7] and humans[8] and knockout of *Fmn2* causes spindle migration failure[7, 9] and impairs the formation of the cytoplasmic actin mesh in oocytes, which is essential for the completion of meiosis I[28, 29]. Formin-2 is known to interact and cooperate with the actin nucleator Spire in oocytes [10, 30–32]. The formin mDia2, one of the isoforms of the mDia family, is localized to the spindle poles in mouse oocytes[33, 34], together with gamma-tubulin, indicating its putative role in meiotic spindle formation; however, the exact role of mDia2 in oocyte maturation has not been characterized yet. In starfish oocytes, the mDia family of formins is involved in the formation of the cleavage furrow during polar body formation and their activity is regulated by phosphorylation via the Mos-MAPK kinase pathway[35]. However, the exact roles of the other formins in oocyte maturation remain to be characterized.

In this study, we utilized the recently developed formin antagonist small molecule inhibitor of formin homology 2 domains (SMIFH2)[36] to determine the collective functions of formin proteins in mouse oocyte maturation. SMIFH2 targets the conserved formin-homology 2 (FH2) domain and inhibits all formins of a broad range of species including the mammalian mDia family, the Bni/Bnr family of formins from fission yeast[36], and plant formin AtFH1[37]. Therefore, we used it in this study to collectively inhibit the formin family of proteins in oocytes. In addition to the chemical treatment, we used RNAi to examine the roles of mDia1 or mDia2 formins in oocyte maturation and compared the knockdown phenotypes with those generated by SMIFH2 treatment.

## Materials and Methods

### Antibodies & chemicals

Goat polyclonal antibody against human Dia2 (sc-10894) was obtained from Santa Cruz Biotechnology (Santa Cruz, CA, USA), mouse monoclonal anti-α-tubulin-FITC antibody, Phalloidin–Tetramethylrhodamine B isothiocyanate (TRICT) and anti-lectin-FITC were obtained from Sigma (St Louis, MO, USA), and Alexa Fluor 488-conjugated goat anti-mouse antibody was purchased from Invitrogen (Carlsbad, CA, USA). Mouse monoclonal anti-Tpm3.2 antibody (CG3) [38] was obtained from the Developmental Study Hybridoma Bank at the University of Iowa. Chemicals including SMIFH2, milirone, were purchased from Sigma, unless stated otherwise.

### Oocyte collection and culture

All animal manipulations were approved and conducted according to the guidelines of the Animal Research Committee of Chungbuk National University (approval no. CB-R28).

Germinal vesicle (GV)-intact oocytes were collected from the ovaries of 6–8-week-old Imprinting Control Region (ICR) mice 48h after administration of an injection of 5 IU of Pregnant mare's serum gonadotropin (Daesung biochemical, Daejun, Korea). Mice were sacrificed by cervical dislocation. Oocytes were cultured in M16 medium (Sigma, St.Louis, MO, USA) under paraffin oil at 37°C with 5% CO_2_. Oocytes were collected for immunostaining and microinjection after they had been cultured for various lengths of time. For SMIFH2 treatment, SMIFH2 dissolved in dimethylsulfoxide (DMSO) was added to final concentrations of 100–500 μM. In control- or SMIFH2-treated groups, DMSO concentrations were adjusted to 0.5% w/v, respectively.

### Real-time quantitative PCR analysis

The mRNA levels of mDia1 and mDia2 in mouse oocytes were determined by using real-time quantitative PCR. Total RNA was extracted from 50 oocytes using a Dynabeads mRNA DIRECT Kit (Life Technologies, Foster City, CA, USA). First-strand cDNA was generated using a cDNA Synthesis Kit (Takara, Kyoto, Japan) and oligo(dT) 12–18 primers. The PCR primers used to amplify *mDia1* and *mDia2* are listed in Table 1. Real-time PCR was performed with SYBR Green in a final reaction volume of 20 μl (DyNAmo SYBR Green qPCR Kit; Finnzymes, Vantaa, Finland). PCR conditions were as follows: initial denaturation at 94°C for 10 min, followed by 39 cycles at 95°C for 15 s, 60°C for 15 s, and 72°C for 45 s, and a final extension at 72°C for 5 min. Gene expression was normalized to the level of GAPDH mRNA and quantified by using the ΔΔCT method[39]. Experiments were conducted in triplicate.

**Table 1:** Primers used in this study.

Gene(Accession No.)	Primer sequence	Use of the primer
GAPDH(NC_000072.6)	5’-AACTTTGGCATTGTGGAAGG-3’	qPCR (Forward)
	5’-ACACATTGGGGGTAGGAACA-3’	qPCR (Reverse)
mDia1(NM_007858.2)	5’-CGCCATCCTCTTCAAGCTAC-3’	qPCR (Reverse)
	5’-CACGCAAGAAATGCAACAGA-3’	qPCR (Reverse)
	5’-CACATAATACGACTCACTATAGGGTCCAGCTGAGGAACTGGACT-3’	dsRNA (Forward)
	5’-CACATAATACGACTCACTATAGGGTTTCAGAATCCAAGGCATCC-3’	dsRNA (Reverse)
mDia2(NM_019670.1)	5’-CGGGTGCCATATGAGAAAAT-3’	qPCR (Forward)
	5’-TGACAGCCATGATGTCAGGT-3’	qPCR (Reverse)
	5’- CACATAATACGACTCACTATAGGGGGACTCGGATTATTGCTGGA -3’	dsRNA (Forward)
	5’- CACATAATACGACTCACTATAGGGTGAGCAGGAGATGCTGAAGA-3’	dsRNA (Reverse)
eGFP	5’-ATTAATACGACTAACTATAGGGAGAATGGTGAGCAAGGGCGAG-3’	dsRNA (Forward)
	5’-ATTAATACGACTCACTATAGGGAGAGCTCGTCCATGCCGAGAG-3’	dsRNA (Reverse)

dsRNA; double-stranded RNA; qPCR, quantitative real-time PCR.

### Preparation of double-stranded RNA (dsRNA)

mDia1 and mDia2 dsRNAs were generated as described previously[19]. Briefly, 610 bp of mDia1 (nt 942–1552 of NM_007858.2) and 687 bp of mDia2 (nt 589–1276 of NM_019670.1) were amplified from first-strand cDNA generated from RNA that was extracted from MII oocytes, by using gene-specific primers containing the T7 promoter sequence (Table 1). *In vitro* transcription was performed using a mMESSAGE mMACHINE T7 Kit (ThermoFischer Scientific, Waltham, MA, USA). The dsRNA was treated with DNase I to remove any contaminating DNA, purified by phenol-chloroform extraction and isopropyl alcohol precipitation, and stored at -80°C until use.

### RNA interference (RNAi)

Microinjection of dsRNA in GV-stage mouse oocytes was done as described previously[19, 40]. To knock down mDia1 and/or mDia2, 1 μg/μl mDia1 and/or mDia2 dsRNA were microinjected into the cytoplasm of a fully grown GV-stage oocyte using an Eppendorf FemtoJet (Eppendorf AG, Hamburg, Germany) and a Nikon ECLIPSE TE300 inverted microscope (Nikon UK, Kingston upon Thames, Surrey, UK) equipped with an MM0-202N hydraulic three-dimensional micromanipulator (Narishige, Sea Cliff, NY, USA). After injection, the oocytes were incubated in M16 medium containing 5 μM milrinone (Sigma, St.Louis, MO, USA) and then washed five times in fresh M16 medium for 2 min each time. The oocytes were then transferred to fresh M16 medium and cultured for a further 12 h. The developmental stages of the oocytes were determined by DAPI staining. Control oocytes were microinjected with 5–10 pl of negative control dsRNA against GFP[41]. Polar body extrusion and cytokinesis were observed using a stereo microscope.

### Immunostaining and confocal microscopy

For immunostaining of mDia2 and the microtubules, oocytes were fixed in 4% paraformaldehyde dissolved in phosphate-buffered saline (PBS) and then incubated in a membrane permeabilization solution (0.5% Triton X-100) for 1 h. In the case of of Tpm3-2, mouse oocytes were fixed and permeabilized in methanol at -20°C as previously described[18]. After 1-h incubation in blocking buffer (PBS containing 1% bovine serum albumin), the oocytes were incubated overnight at 4°C with mDia2 primary antibody (1:200 dilution). The oocytes were washed three times with wash buffer (PBS containing 0.1% Tween-20 and 0.01% Triton X-100) and labeled with Alexa Fluor 488-conjugated goat-anti-mouse IgG (1:100 dilution) for 1–2 h at room temperature. To stain the cytoplasmic actin mesh or the cortical actin, the oocytes were fixed and stained with the F-actin stain phalloidin-TRITC (10 μg/ml). For immunostaining of tubulin and cortical granules, oocytes were incubated with anti-lectin-FITC and anti-α-tubulin-FITC (1:200, respectively) for 1 h, washed three times in wash buffer for 2 min, incubated with Hoechst 33342 (10 μg/ml in PBS) for 15 min, and washed three times.

Samples were mounted onto glass slides and examined using a confocal laser scanning microscope (Zeiss LSM 710 META, Jena, Germany using a 40× water immersion objective lens for fixed oocytes and a 63× oil immersion objective lens for cytoplasmic actin mesh staining). The fluorescence intensities of actin labeling were quantified using the ImageJ software [42].

### Data analysis

For each treatment, at least three repeats were performed. Statistical analyses were conducted using Pearson’s chi-square test followed by post-hoc false-discovery rate analysis for p-value correction, or by analysis of variance (ANOVA) followed by Tukey’s multiple comparison test, using R (R Development Core Team, Vienna, Austria). Data are expressed as mean ± standard error of the mean and p < 0.05 was considered significant.

## Results

### Treatment with SMIFH2 inhibits mouse oocyte maturation

**Fig 1:**
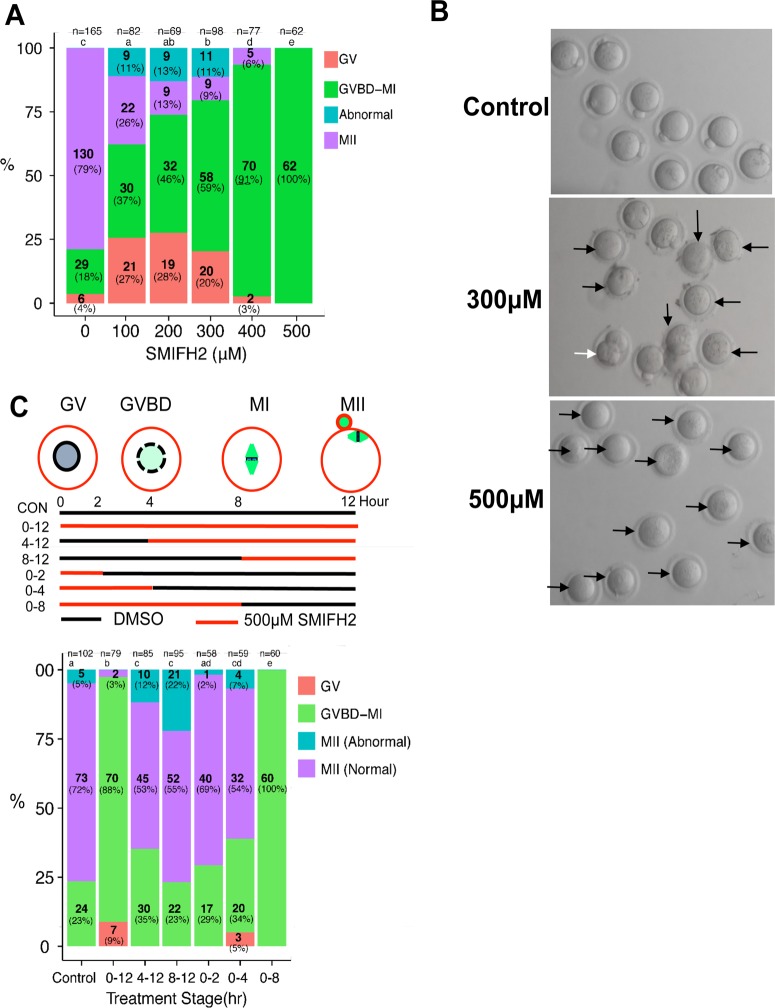
The formin inhibitor SMIFH2 blocks mouse oocyte maturation. **A.** Treatment with SMIFH2 decreases the oocyte maturation rate and increases the ratio of symmetric division. SMIFH2 was added to M16 culture medium at concentrations of 0, 100, 200, 300, 400, and 500 μM and immature oocytes were cultured for 12 h after which their status of development was assessed. At least 3 independent experiments were carried out and the ratios of oocytes at each developmental stage to total oocytes were plotted. Significant differences (p < 0.05) between the treated groups are indicated by a different superscript letters(a,b,c,d and e). **B.** Representative pictures of oocytes treated with SMIFH2. White arrows indicate abnormally matured oocytes, while black arrows mark oocytes that failed to mature at the MII stage after 12 h of growth. **C:** Treatment with SMIFH2 for distinct durations and the effect thereof on oocyte maturation. CON: control group without SMIFH2 treatment; 0–12: the oocytes were treated with 500 μM of SMIFH2 during the entire incubation time; 0–2, 0–4, 0–8: the oocytes were treated with 500 μM of SMIFH2 during 0–2, 0–4, and 0–8 h after the start of incubation, respectively, and then transferred to fresh M16 medium without SMIFH2; 4–12, 8–12: the oocytes were cultured without SMIFH2 for 4 and 8 h, respectively, after which 500 μM of SMIFH2 was added to the medium. Maturation ratios after 12 h of incubation for each treatment group are plotted. Statistical differences (p < 0.05) between different treatments are indicated by a different superscript letters(a,b,c,d and e).

We treated mouse oocytes with 100–500 μM of SMIFH2 during *in vitro* maturation (IVM) and examined the effects on oocyte maturation. As shown in Fig. 1A, treatment with 100 μM SMIFH2 significantly decreased oocyte maturation at the metaphase II (MII) compared to the control, while treatment with 500 μM completely blocked oocyte maturation at this stage. In the range of 100–300 μM SMIFH2, some oocytes completed cytokinesis (20% to 36%), but even oocytes with complete cytokinesis usually failed to undergo asymmetric division. In addition, the ratio of oocytes with abnormal division, defined as oocytes having a polar body with a diameter greater than 50% of that of the oocyte, was increased to 30–50% in oocytes that completed cytokinesis after treatment with 100–300mM of SMIFH2 (Fig. 1A and 1B), indicating that the spindle migration was impaired.

Next, we tested the effect of different SMIFH2 treatment durations on the maturation rate. As shown in Fig. 1C, while maturation was nearly completely inhibited during the whole course of IVM (0–12 h) in the presence of 500 μM of SMIFH2, treatment of SMIFH2 during 0–4 h and replace with SMIFH2-free medium restored the maturation rate to 60% (indicated as 0-4h in Fig. 1C), indicating that germinal vesicle breakdown (GVBD) itself was not affected by SMIFH2 treatment. Maturation was nearly abolished after treatment with SMIFH2 for 0–8 h followed by continued maturation without SMIFH2, indicating that SMIFH2 treatment after GVBD is crucial for full inhibition of oocyte maturation. Finally, when SMIFH2 was added to the culture medium for 4–12 h or 8–12 h after the start of IVM, the maturation rates were not affected, although the asymmetric division rates of these groups were increased compared to that of the control. These results suggested that the inhibitory effect of SMIFH2 on oocyte maturation takes effect after the GVBD-MI transition.

### SMIFH2 treatment abrogates spindle formation and actin polymerization

**Fig 2:**
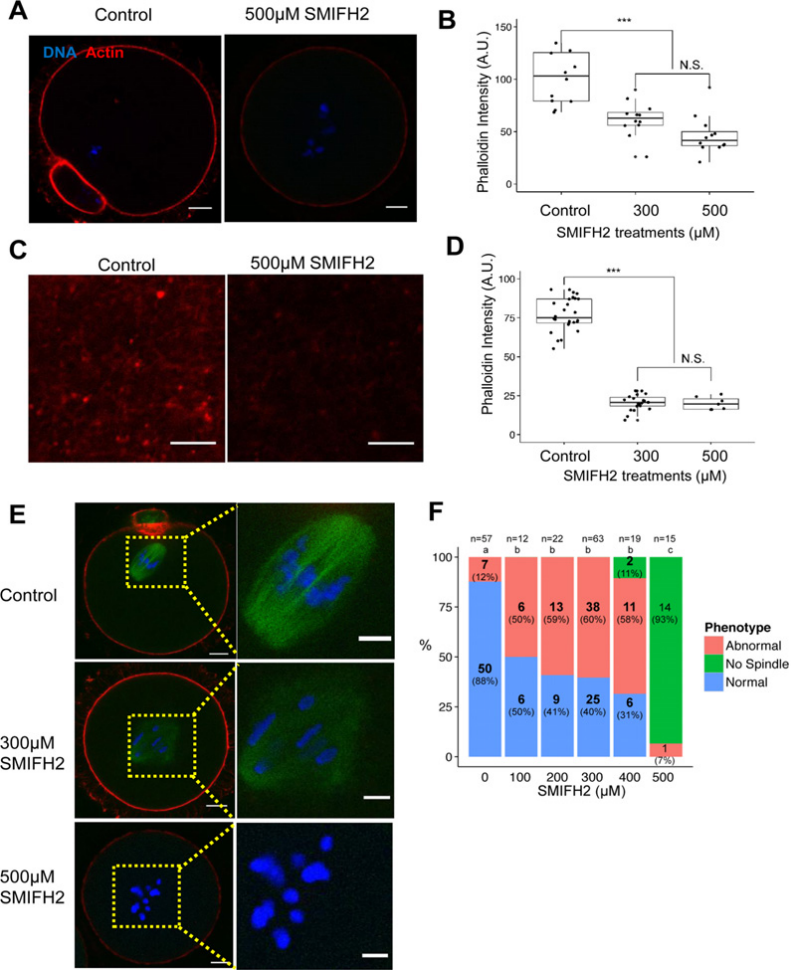
Treatment with SMIFH2 decreases the actin level in oocytes and impairs spindle formation. **A**. Treatment with SMIFH2 decreases the cortical actin level in maturing mouse oocytes. Control (left) and oocytes treated with 500 μM of SMIFH2 were stained with phalloidin to visualize actin (red). Chromatin was stained with Hoechst 33342 (blue). Bar = 10 μm. **B**. Quantification of the cortical actin levels in SMIFH2-treated (300 and 500 μM) and control oocytes. Actin in the cortex regions excluding the cortical actin cap and polar body regions was quantified using ImageJ[42]. The box represents the interquartile range; the whiskers show 1.5x the interquartile range; line inside the box represents the median (control: n = 14; 300 μM: n = 12; 500 μM: n = 12). N.S.: not significant (p > 0.05); ***: significantly different from control oocytes (p < 0.001). **C**. Phalloidin-stained cytoplasmic actin mesh in oocytes. Untreated (control) oocytes and oocytes treated with 500 μM of SMIFH2 were stained with phalloidin to visualize actin (red). Bar = 3 μm. **D.** Quantification of the cytoplasmic actin stained with phalloidin in control (n = 27) and SMIFH2-treated (300 μM: n = 21; 500 μM: n = 7) cells. **E.** Abnormal spindle formation induced by SMIFH2 treatment. Spindles of control or SMIFH2-treated (300 and 500 μM) oocytes were visualized by immunostaining with anti-α-tubulin (green), and chromatin was stained with Hoechst 33342 (blue). No spindle structures are detected in oocytes treated with 500 μM of SMIFH2. Bar: 10 μm. **F.** Effect of various SMIFH2 concentrations on spindle formation and morphology. Oocytes were classified based on the spindle morphology. Normal: oocytes with normal spindle shape with proper chromatin alignment, similar to the spindle shape of the control cells in panel E; Abnormal: oocytes containing a spindle with improper chromatin alignment or irregular shape, similar to that of cells treated with 300 μM SMIFH2 in panel E; No spindle: oocytes with no detectable spindle structure, similar to that of cells treated with 500 μM SMIFH2. The ratio of each spindle type of oocytes to total oocytes was plotted. Significant differences (p < 0.05) between different treatments are indicated by a different superscript letter(a,b and c).

To investigate the exact mechanism of SMIFH2-mediated inhibition of oocyte maturation, we examined the distribution of the actin filaments and microtubules in SMIFH2-treated and control oocytes by using immunostaining. As shown in Fig. 2A and 2B, the amount of cortical actin decreased significantly in the SMIFH2-treated groups, confirming that formins play a role in establishing the cortical actin in oocytes. In addition, we measured the amount of actin within the cytoplasmic actin meshwork in control and SMIFH2-treated cells, which showed that SMIFH2 effectively decreased the actin mesh levels (Fig. 2C and 2D), confirmed the previous reports[10, 28, 32] that cytoplasmic actin mesh in oocytes was generated by formin-2.

Staining of the microtubules showed that treatment with 100–400 μM of SMIFH2 increased the formation of spindles with abnormal morphology compared to the control treatment (Fig. 2E and 2F). Treatment with 500 μM of SMIFH2 abolished spindle formation in 93% of the oocytes. In most cases, the chromatin devoid of microtubules in SMIFH2 treated-oocytes remained in the center of oocyte. Collectively, these results suggested that treatment with SMIFH2 affects both cortical actin and meiotic spindle formation.

### SMIFH2 treatment impairs the formation of the cortical granule-free domain and decreases the amount of tropomyosin

**Fig 3:**
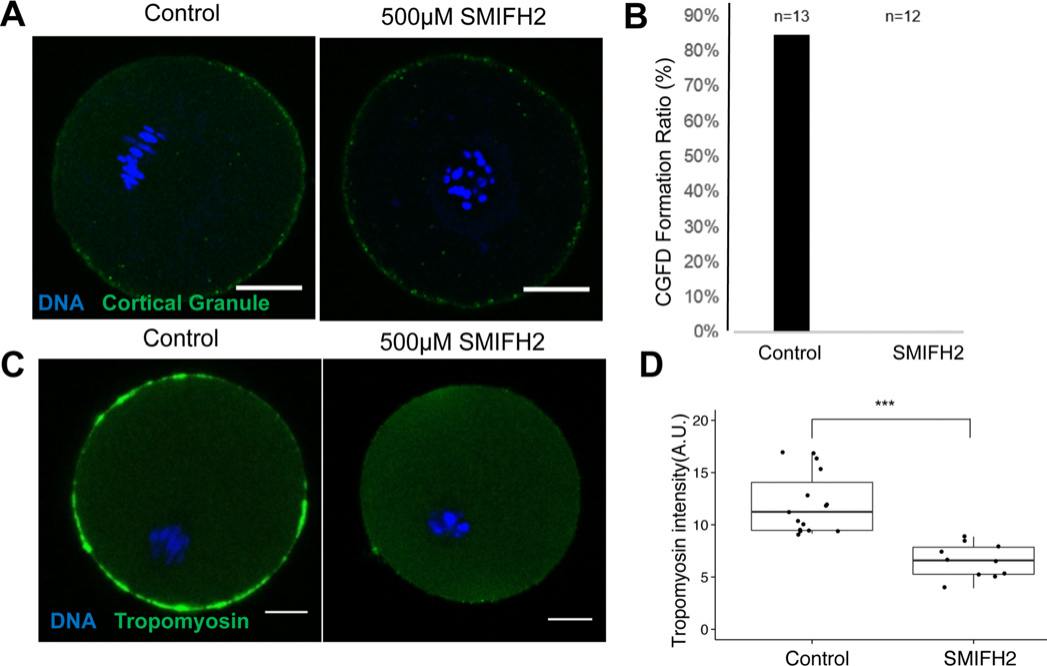
Treatment with SMIFH2 ablates formation of the cortical granule free domain (CGFD) and decreases the cortical tropomyosin levels. **A.** Treatment with SMIFH2 impairs the formation of the cortical granule-free domain (CGFD) in comparison to control oocytes (left). Oocytes treated with 500 μM of SMIFH2 failed to form the CGFD, which is stained with labeled lectin (green). Chromatin was visualized with Hoechst 33342 (blue). Bar: 20 μm. **B.** CGFD formation (%) in control oocytes (n = 13) and in oocytes treated with 500 μM of SMIFH2 (n = 12). **C.** Treatment with SMIFH2 affects the cortical tropomyosin levels. In control oocytes (left) and oocytes treated with 500 μM of SMIFH2, Tpm3-2 in cortical actin was immunostained (green) and DNA was stained with Hoechst 33342 (blue). Bar: 20 μm. **D.** Intensity of the cortical tropomyosin in control oocytes (n = 17) and in oocytes treated with 500 μM of SMIFH2 (n = 10). The box represents the interquartile range; the whiskers show 1.5x the interquartile range; line inside the box represents the median.

Oocytes in which the chromatin failed to migrate to near the cortex upon SMIFH2 treatment, failed to form the cortical granule-free domain (CGFD) (Fig. 3a and 3b). We investigated the localization of non-muscle isoform of tropomyosin 3 (Tpm3-2), which has been reported to co-localize with cortical actin during the GV, GVBD, and MI stages of mouse oocyte maturation and knockdown of it caused abnormalities in oocyte maturation [18]. Upon treatment with SMIFH2, the amount of Tpm3 in the cortex decreased significantly (Fig. 3c and 3d), indicating that actin filaments decorated by tropomyosins are mainly generated from formins in mouse oocytes.

### Knockdown of mDia1 or mDia2 causes decreased oocyte maturation and abnormal spindle morphology

The abrogation of spindle formation by treatment with SMIFH2 suggested that the formins are involved in meiotic spindle formation in oocytes. It has previously been reported that mDia formins bind to microtubules[43] and stabilize them[44–46]. In addition, it has been shown that the microtubule stabilization activity of mDia formins is independent from their actin polymerization activity[46]. In mammalian oocytes, mDia1 and mDia2 have been reported to localize to the meiotic spindle, particularly to the spindle poles in the case of mDia2[33, 34]. As the oocytes from Fmn2 (-/-) knockout mice can form normal meiotic spindles[7, 9], we hypothesized that the impairment of spindle formation by SMIFH2 is caused by the inhibition of formins other than Formin-2. Based on previous reports of the relationship between mDia formins and microtubule stability[43–46] and of their localization to meiotic spindles, we tested whether the mDia formins are involved in meiotic spindle formation.

**Fig 4:**
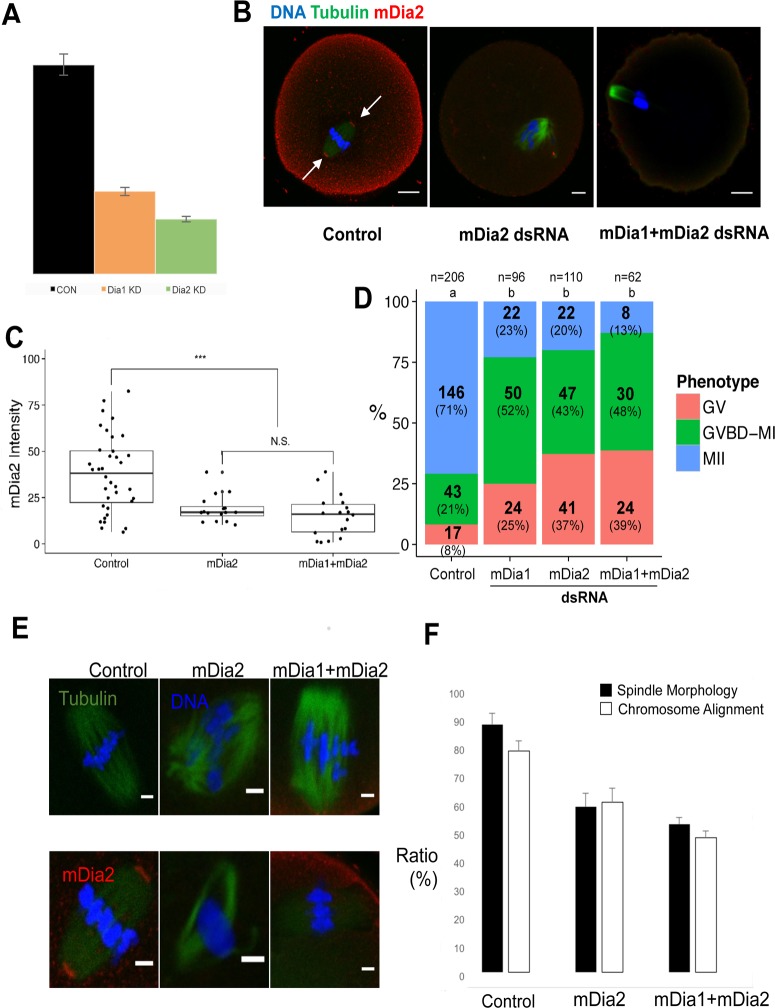
Knockdown of mDia formins impairs oocyte maturation and spindle formation. **A.** The RNA levels of mDia1 and mDia2 in oocytes injected with dsRNAs against mDia1 and mDia2. The expression levels are expressed relative to the expression level in control oocytes treated with control dsRNA. **B.** Morphology and mDia2 localization in oocytes injected with control, mDia2, or mDia1 + mDia2 dsRNAs. Red: mDia2; green: α-tubulin; blue: DNA. White arrows indicate mDia2 located at the spindle poles in control oocytes. **C.** Quantification of mDia2 in oocytes injected with control, mDia2, or mDia1 + mDia2 dsRNAs. ***: significantly different from the negative control dsRNA-injected oocytes (p < 0.001); N.S.: not significant (p > 0.05). The boxes show the interquartile range; the whiskers show the 1.5 × the interquartile range; the line inside the box represents the median. **D.** Knockdown of mDia1, mDia2, or mDia1 + mDia2 decreases the oocyte maturation rate and increases the symmetric division rate. dsRNA against mDia1, mDia2, or a mixture of mDia1 and mDia2 were microinjected into immature oocytes along with control dsRNA and cells were cultured for 12 h after which their status of development was assessed. Significant differences (p < 0.05) between different treatments are indicated by a different superscript letter. **E.** Knockdown of mDia2 or mDia1 + mDia2 impairs spindle formation. Oocytes injected with mDia2, mDia1 + mDia2, or control dsRNA were incubated for 9 h and their spindle morphology was assessed after immunostaining for alpha-tubulin (green), chromatin (blue), and mDia2 (red). **F.** Percentage of oocytes with a normal spindle after injection with Dia2, mDia1 + mDia2, or control dsRNA. The filled bar represents the oocytes that had a spindle with normal morphology, while the white bar represents the oocytes that displayed normal chromosome alignment in spindle. Each experiment was performed in triplicate and the mean ratios of cells with normal spindles and chromosome alignment were plotted. Error bars indicate the standard error of the mean (SEM). ***: significantly different (p < 0.005); N.S.: not significant (p > 0.05).

To ablate the mDia family formins, we designed dsRNA against mouse mDia1 and mDia2. As shown in Fig. 4a, microinjection of oocytes with dsRNAs against mDia1 and mDia2 effectively decreased their mRNA levels to 39% and 26%, respectively, compared to those in the controls. We checked the protein levels of mDia2 using immunostaining and western blotting. As shown in Fig. 4b and 4c, the mDia2 protein level significantly decreased by mDia2 knockdown.

We evaluated the effects of mDia1 and mDia2 knockdown on oocyte maturation. the maturation rate significantly decreased after knockdown of mDia1 or mDia2 compared to the control(Fig. 4d), indicating that both proteins are required for the asymmetric division in the oocytes. However, simultaneous knockdown of mDia1 and mDia2 did not decrease the maturation rate further. The cortical and cytoplasmic actin levels in mDia2 and mDia1 + mDia2 knockdown groups did not differ from those in the control group (S1 Fig.).

Next, we examined the effect of mDia knockdown on spindle formation. Interestingly, knockdown of mDia1 did not affect spindle formation (data not shown), while knockdown of mDia2 caused abnormal spindle formation (Fig. 4E). In the control oocytes, mDia2 localized to the spindle poles, as previously reported[33]. When mDia2 was knocked down, mDia2 no longer localized to the spindle and abnormal spindle morphology was observed in 40–50% of the oocytes (Fig. 4F). These results indicated that mDia2 is necessary for the formation of a proper meiotic spindle.

## Discussion

In this study, we investigated the roles of the formin family of proteins using two complementary approaches. Previously, the roles of Formin-2 in oocyte maturation have been extensively studied[9, 10, 29, 33, 47, 48], but the roles of the other 14 formins including mDia1 and mDia2 have not been thoroughly investigated. Because multiple homologs and isoforms of the formins are present in mice, it is not feasible to create a genetic mutant that is completely devoid of all of formins. Therefore, we employed the novel chemical inhibitors SMIFH2 to inhibit the broad range of formins that exist in oocytes.

Our results clearly indicated that the proteins of the formin family are crucial for oocyte maturation. Treatments of SMIFH2 caused two different phenotypes, depend on concentrations. In lower (100–300μM) concentrations of SMIFH2 treatments, oocytes failed to complete cytokinesis have been increased significantly. Because formin-2 has been known to be essential for spindle migrations[7, 9] and decreased cortical actin and cytoplasmic actin mesh supported the inhibitions of formin family proteins, including formin-2. Interestingly, treatments of SMIFH2 cause decreased localization of nonmuscle tropomyosin (Tpm3.2), which is recently reported to involved in maintenance of cortical integrity during asymmetric division of oocytes[18]. These results suggested that tropomyosin decorated actin filaments are mainly generated from formin family of proteins. Considering recent reports that cortical tension of oocytes play important roles in spindle migrations[15, 49, 50], tropomyosin and formin may be involved in the controlling of cortical tension during oocyte maturation, but their involvements and molecular mechanism remained to be investigated. [49, 50]

Interestingly, the SMIFH2 treatment did not completely mimic the Fmn2 (-/-) oocyte phenotype. In Fmn2 (-/-) oocytes, meiotic spindle formation is not affected, while migration of the spindle and the late stages of cytokinesis are ablated[7, 9]. In SMIFH2-treated oocytes, spindle formation was severely affected. These results suggested that formins other than Formin-2 play a role in spindle formation.

It is noteworthy that treatment with SMIFH2 for 8 h, which generally corresponds with the time needed for the cells to reach the transition to MI stages during oocyte maturation, was sufficient to block oocyte maturation completely and oocytes sampled at this stage showed germinal vesicle breakdown, while spindle formation was entirely blocked. Usually, meiotic spindle generation starts immediately after GVBD, which is around 4 h after the start of meiotic maturation, and it takes an additional 3–4 h to complete spindle formation[51]. Our results showed that SMIFH2 treatment in this stage blocks meiotic spindle formation, suggesting a role for formin family proteins in meiotic spindle formation.

Although formins were characterized as actin nucleation and elongation factors essential for the formation of straight actin filament bundles[22], recently, novel, non-canonical functions as regulators of microtubules[43–46], especially for the mDia formins, have emerged. We showed that knockdown of mDia2 by RNAi affected spindle formation in the oocytes, although the effect was not as drastic as that of SMIFH2 treatment. These results imply that formins besides the mDia family are involved in spindle formation and maintenance. Recent report also showed that knockdown of mDia1 in mouse oocyte impair functional asymmetric division of oocyte and results in abnormal spindle morphology[52] is concord with our results. In this study, we showed that mDia2 is also important for oocyte maturation and proper spindle formations, in addition with mDia1. Further studies using conditional knockout strains for mDia family formins would clarify the roles of mDia in oocyte maturation, because mDia2 (-/-) knockout mouse cannot survive after E12.5 day[53], therefore assessment of fertility in the strain would not be possible.

Then the question rose how SMIFH2 inhibits spindle formation by inhibiting mDia and/or other proteins of the formin family. It is known that SMIFH2 targets the FH2 domain of formins with a half-maximal inhibitor concentration of 5–15 μM [36]. The core FH2 domain is responsible for the actin nucleation and elongation activity of the formins[22]. In addition, the FH2 domain can bind to microtubules[54]. Therefore, it is possible that SMIFH2 interferes with the interaction between microtubules and FH2, thereby affecting the stabilization ability of the formins. It has been reported that microtubule stabilization depends on two microtubule-binding proteins called adenomatous polyposis coli (APC) and end-binding protein 1 (EB1)[45]. Interestingly, APC displays actin-nucleation activity and interacts with mDia1 via association of its C-terminal basic domain with the C-terminal Diaphanous autoregulatory domain (DAD) of mDia1[55]. Therefore, SMIFH2 may interfere with the interaction between APC or EB1 and the mDia formins, inhibiting their microtubule stabilization activity. Because the complex structure between SMIFH2 and the FH2 domain has not been reported, it is difficult to reveal the exact mechanism of SMIFH2-mediated inhibition of meiotic spindle formation.

It is possible that other formin family proteins are involved in the meiotic maturation of oocytes, as mDia2 knockdown partially phenocopied the effects of the SMIFH2 treatment. Further investigation of the roles of formin proteins besides the mDia family in oocyte maturation will shed light on their function in asymmetric division and meiotic spindle formation.

## Supporting Information

S1 Fig Knockdown of mDia1 or mDia2 did not affect the cortical or cytoplasmic actin levels. **A**. Quantification of the cortical actin levels in oocytes injected with mDia2 or mDia1 + mDia2, and in control oocytes. The actin in the cortex regions excluding the cortical actin cap and polar body regions were quantified using ImageJ[42]. N.S.: not significant (p > 0.05).). The boxes show the interquartile range; the whiskers show the 1.5 × the interquartile range; the line inside the box represents the median. The box represents (control: n = 36; mDia2 knockdown: n = 26; mDia1 + mDia2 knockdown: n = 23). N.S.: not significant (p > 0.05). **B**. Quantification of the cytoplasmic actin mesh stained with phalloidin in control oocytes (n = 12) and in oocytes treated with dsRNA (mDia2: n = 6; mDia1+mDia2: n = 11). N.S.: not statistically significant (p > 0.05). The boxes show the interquartile range; the whiskers show the 1.5 × the interquartile range; the line inside the box represents the median.
(TIF)
