# Supplementary figures and images for: Small Molecule Inhibitor of Formin Homology 2 Domains (SMIFH2) Reveals the Roles of the Formin Family of Proteins in Spindle Assembly and Asymmetric Division in Mouse Oocytes
Source: PLoS One. 2015 Apr 2;10(4):e0123438. doi: 10.1371/journal.pone.0123438 (PMC4383420; doi:10.1371/journal.pone.0123438)

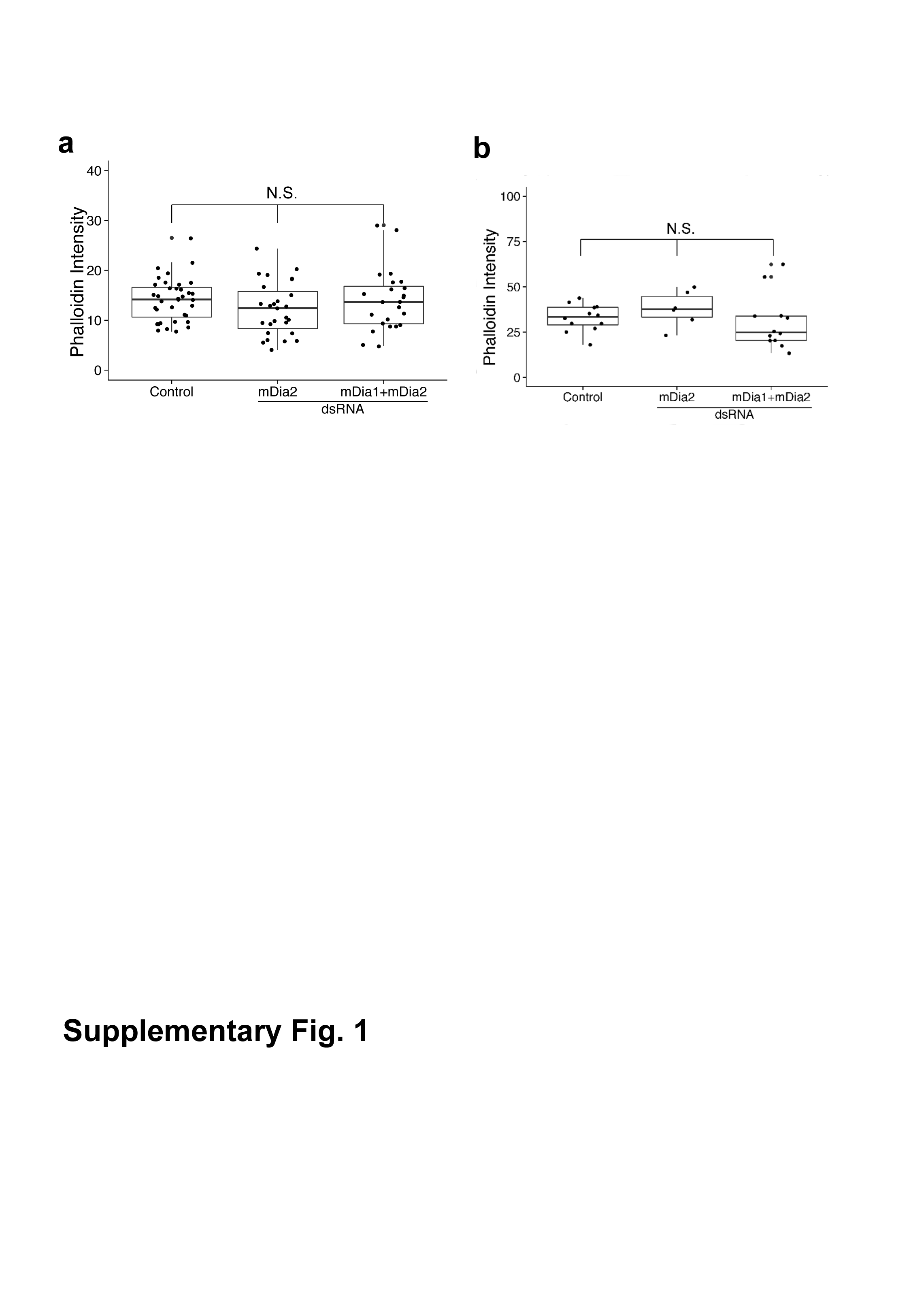

Supplement: S1 Fig — A. Quantification of the cortical actin levels in oocytes injected with mDia2 or mDia1 + mDia2, and in control oocytes. The actin in the cortex regions excluding the cortical actin cap and polar body regions were quantified using ImageJ[42]. N.S.: not significant (p > 0.05).). The boxes show the interquartile range; the whiskers show the 1.5 × the interquartile range; the line inside the box represents the median. The box represents (control: n = 36; mDia2 knockdown: n = 26; mDia1 + mDia2 knockdown: n = 23). N.S.: not significant (p > 0.05). B. Quantification of the cytoplasmic actin mesh stained with phalloidin in control oocytes (n = 12) and in oocytes treated with dsRNA (mDia2: n = 6; mDia1+mDia2: n = 11). N.S.: not statistically significant (p > 0.05). The boxes show the interquartile range; the whiskers show the 1.5 × the interquartile range; the line inside the box represents the median. (TIF) [file pone.0123438.s001.tif]
